# The characteristics of Polish patients with salivary gland tumors: a ten-year single-center experience

**DOI:** 10.1007/s00784-023-05396-2

**Published:** 2023-12-20

**Authors:** Aldona Chloupek, Dariusz Jurkiewicz, Joanna Kania

**Affiliations:** 1 Department of Cranio-Maxillofacial Surgery, Military Institute of Medicine—National Research Institute, 04-142 Warsaw, Poland; 2 Department of Otolaryngology and Oncology, Military Institute of Medicine—National Research Institute, 04-142 Warsaw, Poland; 3 Department of Pathology, Military Institute of Medicine—National Research Institute, 04-142 Warsaw, Poland

**Keywords:** Salivary gland pathologies, Salivary gland tumors, Salivary gland inflammation, Salivary gland surgery

## Abstract

**Objectives:**

The study aims to provide insights into the characteristics of Polish patients with different salivary gland pathologies.

**Materials and methods:**

This is a retrospective study conducted at a single center, including patients who underwent surgery for salivary gland pathologies between 2012 and 2022.

**Results:**

This study included 239 patients who underwent surgery for salivary gland tumors or inflammatory diseases. Malignant tumors were diagnosed in 9.8% of participants, while 64% had benign tumors and 21% suffered from inflammation. The occurrence of complications after surgery was relatively low, with 9.9% of participants experiencing slight facial weakness or mild dysfunction, and 3% experiencing complete paralysis of the facial nerves. Significant differences were observed between patients with cancers and those with benign tumors and inflammation in terms of age. Cancers were more common in females (67% vs. 33%) and predominantly localized in the parotid glands (95%).

**Conclusion:**

Benign tumors, such as Warthin's tumors and polymorphous adenoma, were predominantly found in the parotid glands of patients aged 39–72 years. On the other hand, inflammatory diseases were primarily localized within the submandibular glands of males aged 40–68 years. Additionally, the presence of a malignant tumor was associated with longer hospitalization periods related to surgery and a higher risk of severe complications.

**Clinical relevance:**

This study on Polish patients with salivary gland tumors provides valuable clinical insights that can aid in diagnosis, treatment planning, patient counseling, and further research in the field of oncology. It contributes to the overall understanding of salivary gland tumors, potentially benefiting both patients and healthcare providers.

## Introduction

The salivary glands consist of a diverse group of anatomical structures in which a wide variety of unique pathologies can develop [1]. Based on the WHO histological classification, salivary gland tumors are classified into 13 adenomas and 24 carcinomas [2]. Salivary gland pathologies encompass a spectrum of non-neoplastic disorders, including autoimmune, inflammatory, and infectious conditions, as well as neoplastic disorders, which can be either benign or malignant. These pathologies present with diverse clinical manifestations and exhibit distinct pathomorphological features [3]. Inflammatory and infectious disorders affecting the salivary glands encompass conditions such as sialolithiasis (also known as calculus disease), infectious sialadenitis, inflammatory sialadenitis, and postradiation sialadenitis. These inflammatory diseases, which can manifest as both acute and chronic conditions, are commonly encountered. However, determining their precise prevalence is challenging due to the predominant focus of most epidemiological studies on neoplastic diseases [4]. Many inflammatory salivary glands lesions may mimic neoplastic disease, therefore specialized examinations, including computed tomography or magnetic resonance imaging should be performed to establish the diagnosis.

The incidence of salivary gland tumors varies across different age groups and populations. On average, it is approximately 0.15 cases per 100,000 individuals in patients under 25 years of age, 1.2 cases per 100,000 individuals in those between 25 and 64 years and increases to 4.3 cases per 100,000 individuals in people over 65 years old [5]. Lowest incidence of salivary gland pathologies was reported in Japan, while the highest one in Croatia [6–8]. Tumors of salivary gland account for 3–10% of all head and neck tumors [9–11]. In Europe, their incidence is approximately 8.5% [5]. Apart from exhibiting various histological images, salivary gland tumors also demonstrate variable biological behavior [12]. Pleomorphic adenoma is the most common benign tumor of parotid glands with a malignant transformation potential and odds for recurrence after treatment [13]. Such lesions may be related with cystic change, calcification and hemorrhage [14]. This type of tumor is predominantly composed of ductal, glandular, or solid structures of epithelial elements, while mesenchymal tissues, typically associated with chondroid and fibromyxomatous tissues, are less commonly observed. Due to the ability of pleomorphic adenoma to form small projections invading the surrounding normal parotid tissue, the recurrence rate following tumor enucleation is high, as it can originate from these small projections [9]. Therefore, the recommended approach for the removal of benign parotid gland lesions is partial parotidectomy, which include remove of a tumor with an entire single lobe (superficial or deep) [15, 16]. This method allows for the excision of the lesion without increasing the recurrence rate and is associated with a lower risk of complications compared to total parotidectomy [16]. Clinical reports have indicated that Warthin’s tumors, the second most frequent benign lesion of the parotid gland occur entirely within these glands, usually in the tail of the gland in the form of multiple masses occupying one or both glands [14]. Mucoepidermoid carcinoma (MECa), followed by acinic cell carcinoma (ACCa) and adenoid cystic carcinoma (ADCa) are the most prevalent malignant neoplasms which represent roughly 30% of salivary glands cancers [12]. This type of cancer is localized most commonly in the parotid glands (~ 50%) and minor salivary glands (~ 45%) [14]. Malignant salivary gland cancers are more frequently observed after the 6th decade of life, whereas benign lesions typically present in the 4th to 5th decade of life [12]. The five-year survival of patients with salivary gland neoplasms depends on the stage of disease, but at average, it accounts for about 70% [12]. According to Alvi et al. [12], recognizing the clinical behavior of a tumor is more crucial for treatment planning than histology alone, although histology and grade should also be taken into consideration. In the case of salivary neoplasms, the "rule of 80s" has been observed to hold true. According to this rule, 80% of all salivary tumors occur in the parotid gland, 80% of these tumors are benign, and 80% of these benign tumors in the parotid gland are pleomorphic adenomas [12]. Fine-needle aspiration cytology can be used to diagnose the type of disease, however, such procedure is associated with risk of seeding in case of cancer [17]. As demonstrated above, various salivary gland diseases require different management, therefore, it is very important to make a correct diagnosis.

The aim of this study is to investigate and analyze the demographic and clinical features of Polish patients who have undergone surgical treatment for salivary gland tumors. The study aims to provide insights into the characteristics of these patients and to show differences between patients with different salivary gland pathologies. The epidemiological analysis of salivary gland diseases in adult Poles contained in this article can be used in the diagnosis, prognosis and scheduling of medical services. Moreover, it can be utilized as a basis for further research in this field.

## Materials and methods

### Study design

In this single-centre retrospective study, we collected the data of patients who had undergone surgery for salivary gland pathologies between 2012 and 2022 in the Department of Cranio-Maxillofacial Surgery of Military Institute of Medicine—National Research Institute in Warsaw, Poland.

To determine tumor histology after surgery, the tissue was fixed in 10% buffered formalin for 24 to 72 h and then embedded in paraffin following standard protocols. Sections of 2–3 microns thickness were cut from the paraffin blocks and stained with Hematoxylin and Eosin. The glass slides were digitally scanned using a Panoramic 250 FLASH scanner (3DHISTECH) at 20 × magnification.

All procedures were conducted in accordance with the Declaration of Helsinki. An ethics approval was not required due to the retrospective study design.

### Data collection

The following data were collected and used for the analysis: age, sex, location of condition, tumor histology and grade, duration of hospitalization, and intensity of face paralysis after surgery assessed using House-Brackmann classification score.

### Statistical analysis

Data were analyzed descriptively as medians (interquartile ranges) and counts (percentages). The Kruskal–Wallis rank sum test, Pearson's Chi-squared test and Fisher's exact test were used to compare variables between patients with malignant tumors, mixed tumors, Warthin tumors, and inflammatory diseases. *P*-values < 0.05 were considered statistically significant. The R software (v. 4.2.2) was used for all calculations.

## Results

A total of 239 patients who underwent surgery for salivary gland tumor or inflammatory disease were included in this study. There were slightly more females than males (52% vs. 48%). The mean age of participants was 59 years.

The majority of patients had salivary gland involvement in the parotid glands. Among the participants, 9.8% were diagnosed with malignant tumors, 64% had benign tumors, and 21% suffered from inflammation.

The frequency of complications after surgery was low, with most patients (87%) exhibiting normal facial nerve function in all areas (grade I). Slight facial weakness or other mild dysfunction were observed in 9.9% of participants after surgery. However, a total of 7 patients experienced complete paralysis of the facial nerves.

**Fig. 1:**
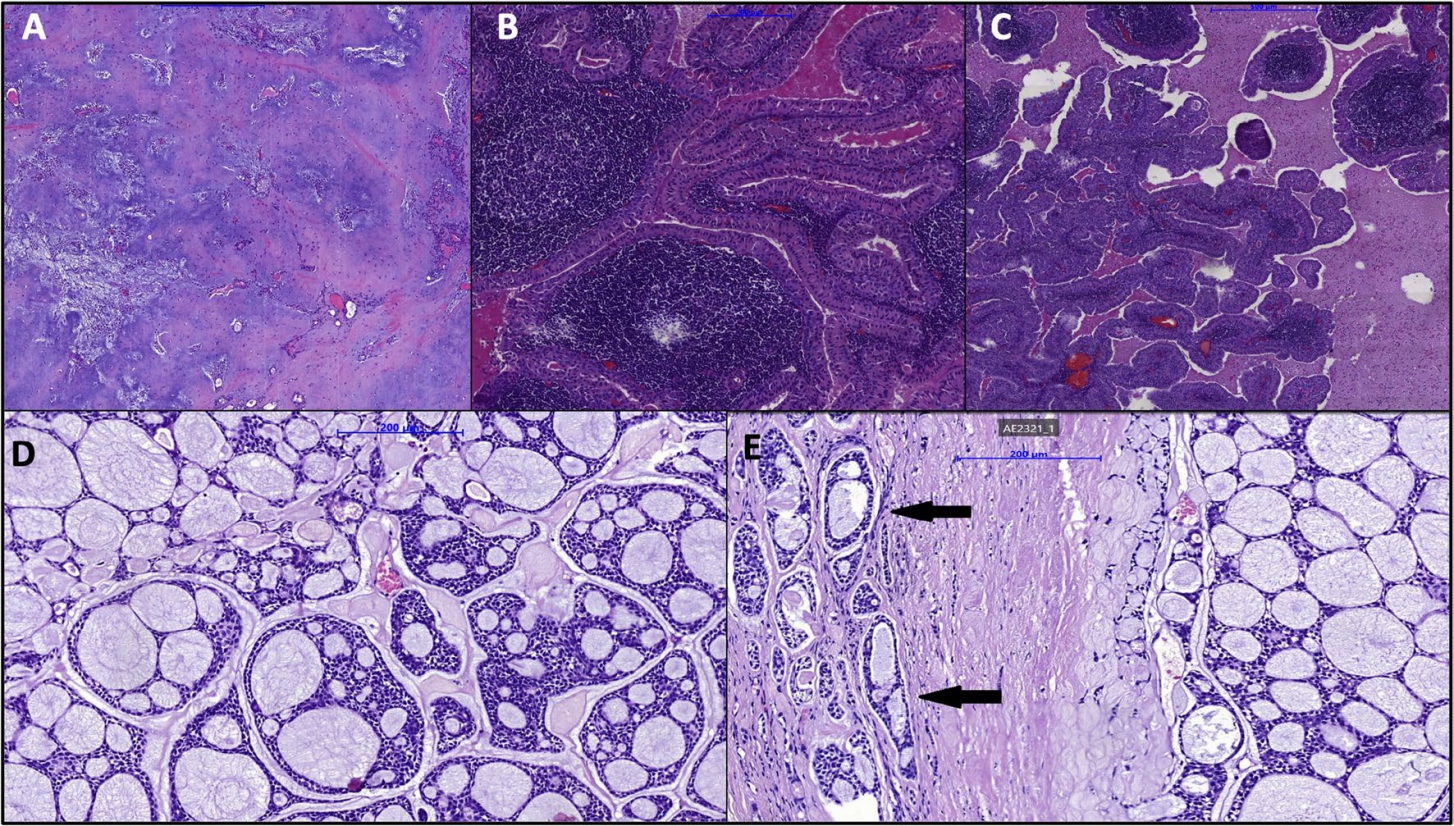
Pathological images of salivary gland tumors. A pleomorphic adenoma—epithelial and myoepithelial cells in chondromyxoid stroma (H&E, × 200); B Warthin tumor (adenolymphoma)—lymphoid stroma with papillary structures lined by oncocytic epithelial cells (H&E, × 200); C Warthin tumor (cystadenolymphoma)—cystic tumor with papillary structures lined by oncocytic epithelial cells and lymphoid stroma (H&E, × 100); D adenoid cystic carcinoma—cribriform pattern (H&E, × 200); E adenoid cystic carcinoma—capsular invasion. Tumor nests (black arrows) invading through the fibrous capsule (H&E, × 200)

The characteristics of the study participants are presented in Table 1. Additionally, Fig. 1 showcases selected pathological images of salivary gland tumors.

**Table 1:** Characteristics of patients

Characteristics	*N* = 239^1^
Age (years)	59 (46, 69)
Sex	
Females (%)	124 (52%)
Males (%)	115 (48%)
Location of the salivary gland	
Sublingual gland (%)	12 (5%)
Submandibular gland (%)	62 (26%)
Parotid glands (%)	165 (69%)
Hospitalization (days)	3.00 (2.00, 4.00)
Histopathological diagnosis	
Malignant tumors (%)	21 (10%)
Pleomorphic adenoma (mixed tumor) (%)	67 (31%)
Warthin tumor (%)	71 (33%)
Inflammation (%)	45 (21%)
Other (%)	10 (5%)
Intensity of facial paralysis after surgery	
Grade I (%)	202 (87%)
Grade II (%)	23 (10%)
Grade VI (%)	7 (3%)

^1^Median (IQR); *n* (%)

**Fig. 2:**
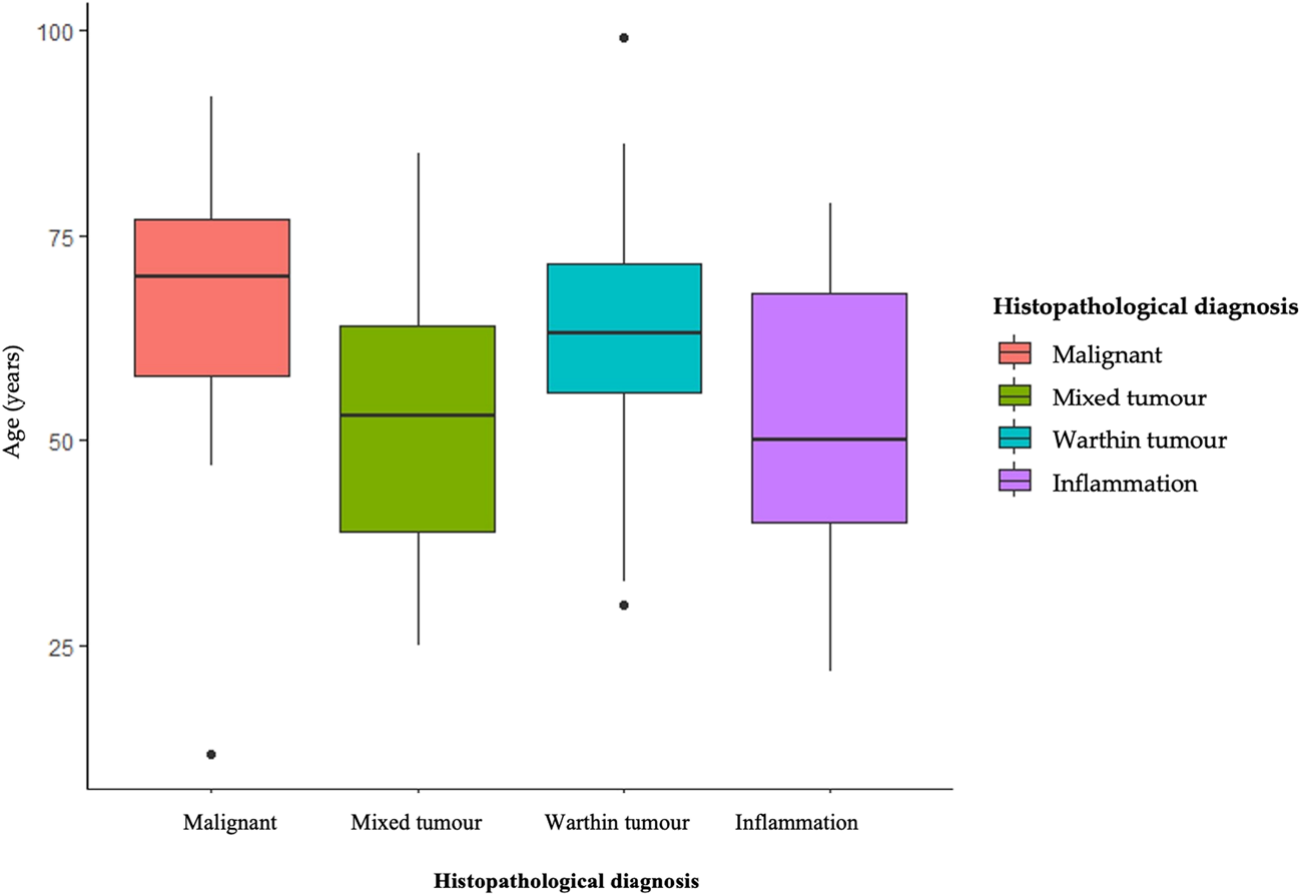
The incidence of different types of parotid glands diseases in various age groups

**Fig. 3:**
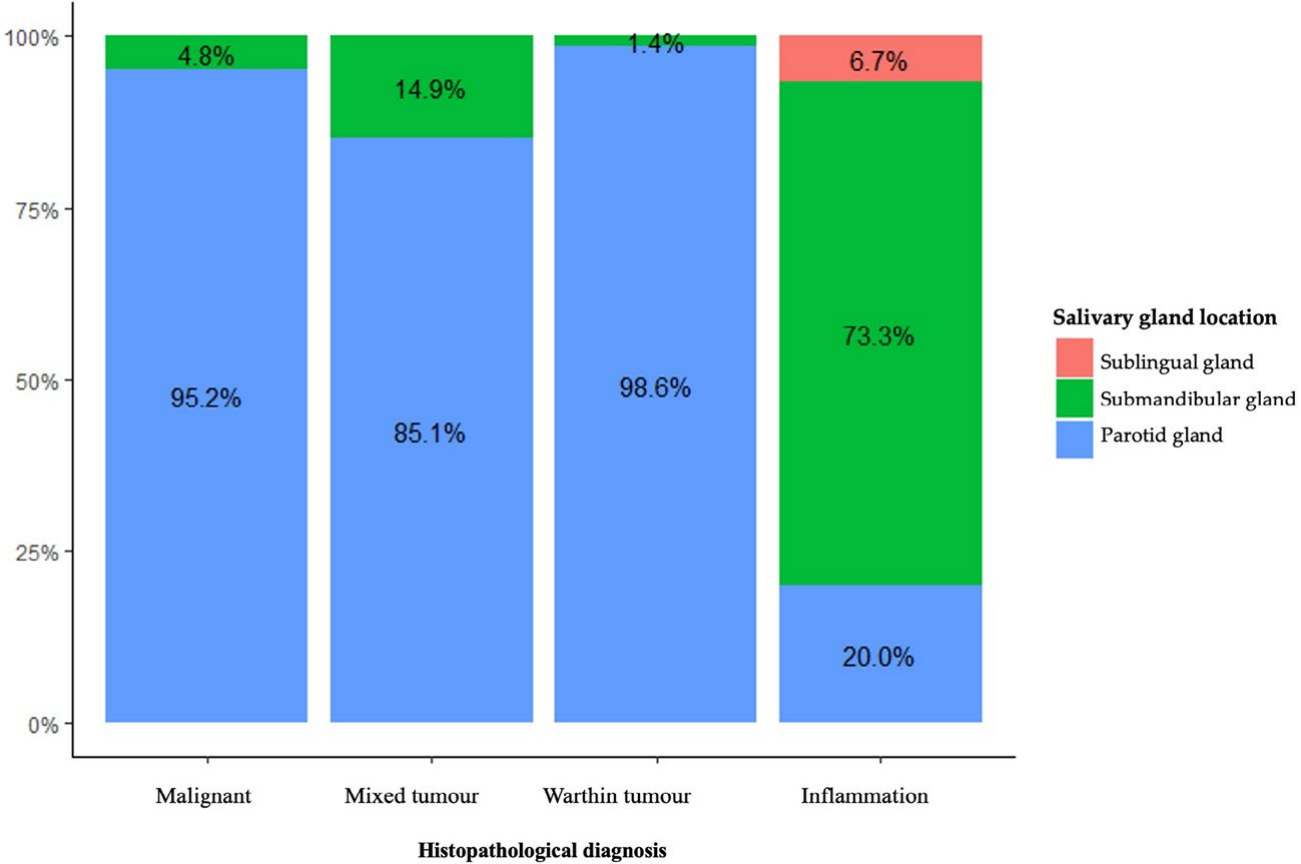
Salivary gland location of analyzed types of diseases

**Fig. 4:**
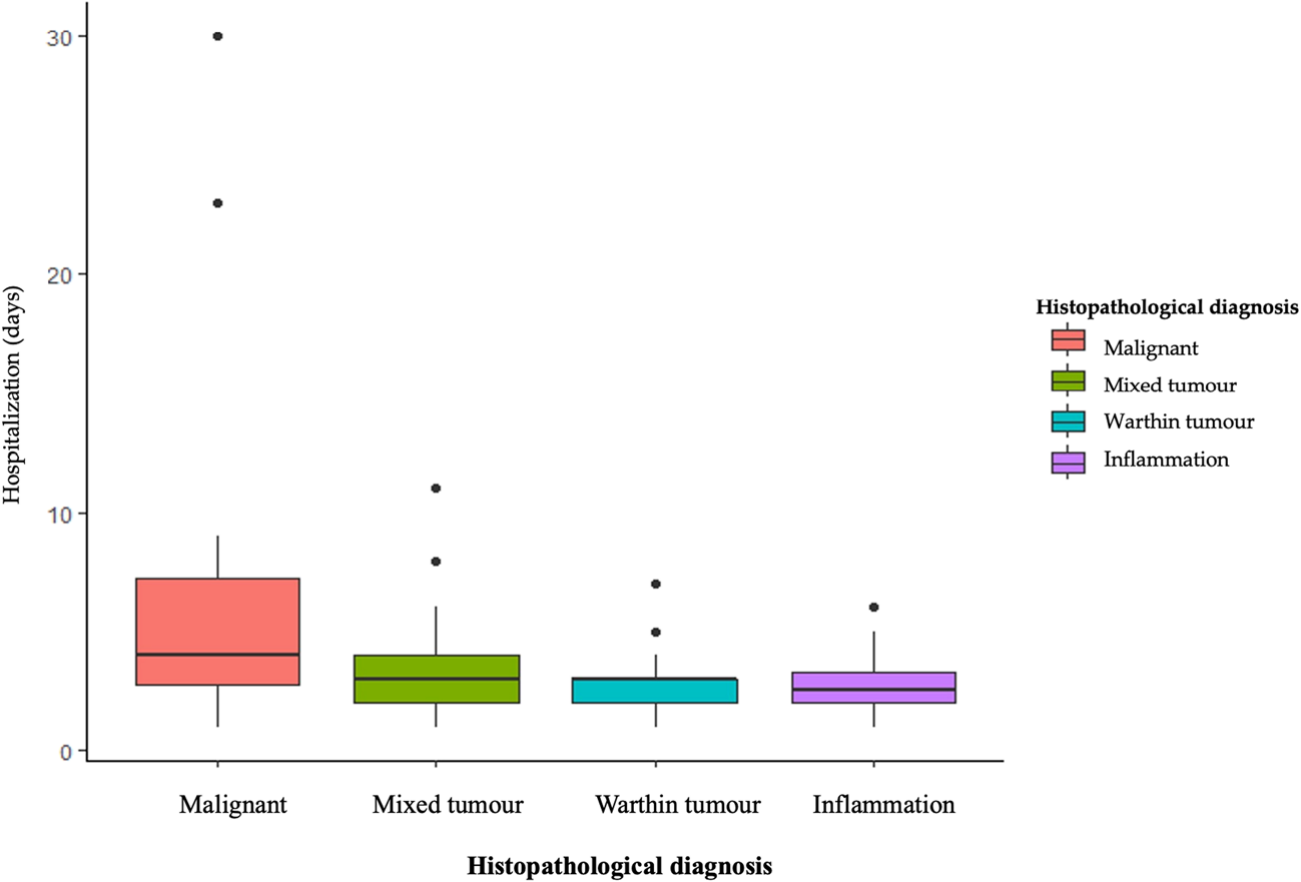
The impact of type of salivary gland disease on the length of hospitalization

Patients with malignant tumors were significantly older compared to those with benign tumors and inflammation (< 0.001) (Fig. 2). Moreover, malignant lesions were more frequent in females compared to males (67% vs. 33%), while males exhibited a higher occurrence of inflammatory diseases. Mixed tumors were also more commonly diagnosed in females compared to males (66% vs. 34%). Inflammation occurred mostly in submandibular glands (73%). Cancers were frequently localised in parotid glands (95%) (Fig. 3). In these glands also mixed tumors and Warthin tumors were frequently detected. The length of hospitalization was significantly higher in case of patients with malicious neoplasms compared to other types of lesions (*p* = 0.013) (Fig. 4). Among patients with malignant tumors, the highest rate of surgical complications was observed, with 15% classified as grade II and 20% as grade VI. On the other hand, the presence of inflammation was associated with the lowest risk of complications after surgery. The characteristics of patients according to disease type are summarized in Table 2.

**Table 2:** Characteristics of patients by tumor type

Characteristic	Malignant, *N* = 21^1^	Mixed tumor, *N* = 67^1^	Warthin tumor, *N* = 71^1^	Inflammation, *N* = 45^1^	*p*^2^
Age	70 (58, 77)	53 (39, 64)	63 (56, 72)	50 (40, 68)	< 0.001
Sex					0.003
Females (%)	14 (67%)	44 (66%)	30 (42%)	16 (36%)	
Males (%)	7 (33%)	23 (34%)	41 (58%)	29 (64%)	
Salivary gland location					< 0.001
Sublingual gland (%)	0 (0%)	0 (0%)	0 (0%)	3 (7%)	
Submandibular gland (%)	1 (5%)	10 (15%)	1 (1%)	33 (73%)	
Parotid gland (%)	20 (95%)	57 (85%)	70 (99%)	9 (20%)	
Hospitalization (days)	4.00 (2.75, 7.25)	3.00 (2.00, 4.00)	3.00 (2.00, 3.00)	2.50 (2.00, 3.25)	0.013
Intensity of facial paralysis after surgery					0.003
Grade I (%)	13 (65%)	56 (85%)	61 (87%)	42 (95%)	
Grade II (%)	3 (15%)	10 (15%)	7 (10%)	2 (5%)	
Grade VI (%)	4 (20%)	0 (0%)	2 (3%)	0 (0%)	

^1^ Median (IQR); *n* (%)

^2^ Kruskal–Wallis rank sum test; Pearson's Chi-squared test; Fisher's exact test “other” category was excluding, missing values were omitted

Facial paralysis after surgery were assessed using House-Brackmann classification score (Grade I: Lack of facial paralysis)

## Discussion

This study aimed to analyze the demographics and clinical features of Polish patients who underwent surgical treatment for salivary gland tumors or inflammatory diseases, providing insights into their characteristics. The study participants consisted of slightly more females than males, with a distribution of 52% and 48%, respectively. Our findings align with the suggestion by the WHO that salivary gland diseases may be more prevalent in females. However, it is important to note that this pattern may vary when analyzing specific types of tumors [18]. In our study, the mean age of participants was 59 years. A retrospective analysis of data from Polish population revealed that salivary gland diseases occurred most frequently in women aged 51–60 years [19]. Furthermore, in our study, patients with malignant cancers were significantly older compared to those with benign tumors and inflammatory lesions. Żurek et al. [19] observed the annual increase in the mean age of onset for benign and malignant lesions in salivary glands. They also noted a decreasing trend for malignant tumors (from 2.2 to 1.4) between 2010 and 2019, and an increasing trend for benign salivary gland neoplasms (from 5.4 to 8.2) [19]. A similar trend was demonstrated in another study involving the Polish population [20]. This finding may be associated with earlier diagnosis and treatment of benign lesions with the potential for malignant transformation [19]. However, the analysis of records (from 2010 to 2021) of patients with salivary gland hospitalized in one of hospitals in Iraq, demonstrated that tumors were most frequent in patients aged 31–40 years, while most infections occurred in the age group 71–80 years [3]. Żurek et al. [19] suggested that age and the affected gland could determine the type of salivary gland disease. The majority of salivary gland tumors are benign, and neoplasms found in the submandibular and sublingual glands are less likely to be malignant. In a retrospective analysis of the Polish National Health Fund, it was observed that benign neoplasms were more prevalent than malignant tumors in patients with salivary gland disorders. This finding aligns with previous studies, which consistently highlight the higher frequency of benign tumors in salivary gland pathology [19]. Numerous studies have consistently shown that the parotid gland is the most susceptible to developing neoplastic lesions [21, 22]. In our study, the majority of patients with neoplasms had affected parotid glands, which is consistent with clinical evidence indicating that approximately 70–85% of salivary gland neoplasms develop in the parotid glands. In contrast, around 10% of neoplasms occur in the sub-mandibular gland, and less than 4% are found in the minor salivary glands [12, 23]. In two other studies conducted in European countries, salivary gland cancers initiated in the parotid gland in 51.8–57.5% of patients [8, 24]. The retrospective analysis of data obtained from the Polish National Health Fund has shown that neoplastic lesions in salivary glands were predominantly found in the parotid glands, with malignant tumors of the parotid gland accounted for 65.3% of all diagnosed cases [19]. However, it is important to note that the parotid gland can also be a frequent site for metastases from squamous cell carcinomas developing in the skin of the head and neck [12]. Mucoepidermoid carcinoma, which is the most common malignant salivary tumor, predominantly occurs in the parotid gland. On the other hand, adenoid cystic carcinoma is more frequently found in the submandibular and minor salivary glands. In our study, mixed tumors were frequently detected in the parotid glands (85%), as well as Warthin tumors (99%). According to literature data, pleomorphic adenoma occurs usually in major and minor salivary glands [25]. Main pathologies in submandibular and parotid glands include infections, sialolithiasis, and mucoceles [26]. In our study, inflammatory diseases were predominantly observed in the submandibular glands (73.3%). According to the available literature, sialadenitis commonly affects the parotid, submandibular, and small salivary glands [27]. Other study indicated that sialolithiasis was more commonly found in major salivary glands, especially in the submandibular and parotid glands [28–30]. According to estimations, about 85% of sialolithiasis develop in the submandibular gland which could be associated with a stagnant flow of saliva in that site and/or the production of mostly mucinous saliva that is viscous and cause more stagnant flow of secretions [31]. Moreover, the saliva produced in submandibular gland is more alkaline, which predisposes to the precipitation of in-organic salts (including calcium and phosphate) and in consequence the formation of salivary stones [32, 33].

In our study, malignant tumors were diagnosed in 9.8% of participants, while 64% of patients had benign tumors and 21% suffered from inflammatory diseases. The statistics reveal that pleomorphic adenoma is the most common benign neoplasm of the parotid gland, whereas mucoepidermoid carcinoma is the most frequent malignant tumor [9]. However, in our study, the incidence of Warthin’s tumor was slightly higher than that of pleomorphic adenoma. Other studies involving Polish population demonstrated that benign salivary gland tumors were usually diagnosed in 55.7–56.5% of patients, while 47.6–49% had malignant tumors [34, 35]. In other countries, the percentage of women with malignant lesions of the salivary glands varies from 46 to 52% [36–39]. The retrospective analysis of data from the Polish National Health Fund revealed that among all parotid neoplasms analyzed, 73.28% of lesions were found to be benign, while 26.72% were identified as malignant. In another retrospective analysis involving 377 patients with parotid gland disease who were scheduled for surgery, 74.5% of the patients had benign parotid gland disease. Among these cases, the most common benign neoplasm was pleomorphic adenoma (40.9%), followed by Warthin's tumor (24.9%), and chronic sialoadenitis (10.7%) [9]. Warthin’s tumor, the second most common benign lesion ac-counted for 24.9% and 22.2% of benign parotid gland disease and all parotid gland disease, respectively [9]. The frequency of Warthin’s tumor in that study was lower compared to our results. In turn, the results of ten-year review of 237 cases demonstrated that Warthin’s tumor was present in 12.7% of patients with benign parotid lesions [40]. The occurrence of this tumor in Chinese population was more common even than pleomorphic adenoma (37% vs. 33%) which resembles the situation in our study [41]. Warthin’s tumor was found to be more prevalent in older patients (average age = 60.6 ± 10.6 years) which is in agreement with our results [9]. Moreover, Chan et al. [9] suggested that in patients with parotid gland tumor who are younger than 45 years, the presence of Warthin’s tumor can be ruled out with high probability. Moreover, they observed increasing incidence of Warthin’s tumor which could be due to prolonged lifespan and the fact that more surgeries are performed on older people [9].

As mentioned earlier, malignant tumors were diagnosed in 9.8% of participants in our study. Similarly, in a retrospective study including patients with salivary gland disease, the malignancy rate was reported as 11.1% [9]. Among the malignant cases, acinic cell carcinoma was the most frequent (22.9%), followed by mucoepidermoid carcinoma (17.1%). Furthermore, when analyzing the incidence of malignant and benign lesions in both genders, our study found that malignant lesions and pleomorphic adenoma were more frequent in females compared to males, while inflammation and Warthin's tumor occurred more frequently in males. In contrast, Chan et al. reported that benign lesions were more common in males (60.1%) compared to females (39.9%) [9]. Similarly to our results, in Chen et al. study, the occurrence of pleomorphic adenoma was higher in females than males (58.3% vs. 41.7%) [9]. They also observed that the most common benign parotid gland disease in male patients was Warthin’s tumor (32.1%), and not pleomorphic adenoma (25.3%) [9]. Moreover, they found that salivary gland diseases occurred more frequently in male patients, especially in the middle-aged group [9]. In contrast, Alvi et al. [12] suggested that benign lesions tend to occur more frequently in females. In turn, malignant lesions appear to develop with equal frequency in both genders [12]. In turn, Żurek et al. [19] failed to observe the association between gender and the type of the pathological lesion.

In this study, we also analyzed the impact of lesion type on the duration of hospitalization. We found that the length of hospitalization was significantly longer for patients with malignant tumors compared to other types of lesions. The frequency of complications after surgery was low in our study. The majority of patients (87%) had normal facial nerve function in all areas (grade I). However, 9.9% of participants experienced slight facial weakness or other mild dysfunction, and 3% of patients showed complete paralysis of the facial nerves. Among the participants, the highest rate of surgery complications was observed in patients with malignant tumors, with 15% classified as grade II and 20% as grade VI. On the other hand, the presence of inflammatory disease was associated with the lowest risk of complications after surgery. Also, Chan et al. [9] found that the most common postoperative complications included temporary facial palsy (20.0%) and permanent facial palsy (2.5%). The risk of temporary facial palsy was similar in patients with benign and malignant tumors in their study. However, permanent facial palsy occurred significantly more frequently in patients with malignant tumors (14.3%). Furthermore, in the retrospective analysis of data collected from patients with salivary gland disease, the most common surgical complications listed were temporary facial palsy (20.0%), permanent facial palsy, hematoma, sialocele, and wound infection [9].

## Conclusions

In summary, our study findings revealed distinct patterns in the occurrence of different salivary gland pathologies. Benign tumors, such as Warthin's tumors and polymorphous adenomas, were predominantly observed in the parotid glands of patients aged 39–72 years. Conversely, inflammatory diseases were primarily localized within the submandibular glands of males aged 40–68 years. Polymorphous adenoma tumors were more common in females, while Warthin's tumors were more frequently observed in males. Similarly, to benign tumors, malignant neoplasms primarily affected the parotid glands. However, their occurrence was higher in older females compared to benign tumors. It is worth noting that the presence of malignant tumors was associated with longer hospitalization periods related to surgery and an increased risk of severe complications.

## Data Availability

The data that support the findings of this study are available from the corresponding author upon reasonable request.
